# Viral and immune factors associated with successful treatment withdrawal in HBeAg-negative chronic hepatitis B patients

**DOI:** 10.1016/j.jhep.2020.11.043

**Published:** 2021-05

**Authors:** Mireia García-López, Sabela Lens, Laura J. Pallett, Barbara Testoni, Sergio Rodríguez-Tajes, Zoe Mariño, Concepción Bartres, Ester García-Pras, Thais Leonel, Elena Perpiñán, Juan José Lozano, Francisco Rodríguez-Frías, George Koutsoudakis, Fabien Zoulim, Mala K. Maini, Xavier Forns, Sofía Pérez-del-Pulgar

**Affiliations:** 1 Liver Unit, Hospital Clínic, University of Barcelona, IDIBAPS, CIBERehd, Barcelona, Spain; 2 Division of Infection and Immunity, Institute of Immunity and Transplantation, University College London, London, UK; 3 INSERM U1052-Cancer Research Center of Lyon (CRCL), University of Lyon, UMR_S1052, CRCL, Lyon, France; 4 Bioinformatics Platform, CIBERehd, Barcelona, Spain; 5 Liver Pathology Unit, Department of Biochemistry and Microbiology, Hospital Universitari Vall d’Hebron, Universitat Autònoma de Barcelona, CIBERehd, Barcelona, Spain

**Keywords:** Hepatitis B virus, Chronic hepatitis B, Nucleos(t)ide analogue, Antiviral therapy discontinuation, HBsAg, HBcrAg, Covalently closed circular DNA, HBV-RNA, Immune response, HBV-Specific T cells

## Abstract

**Background & Aims:**

Factors associated with a successful outcome upon nucleos(t)ide analogue (NA) treatment withdrawal in HBeAg-negative chronic hepatitis B (CHB) patients have yet to be clarified. The objective of this study was to analyse the HBV-specific T cell response, in parallel with peripheral and intrahepatic viral parameters, in patients undergoing NA discontinuation.

**Methods:**

Twenty-seven patients without cirrhosis with HBeAg-negative CHB with complete viral suppression (>3 years) were studied prospectively. Intrahepatic HBV-DNA (iHBV-DNA), intrahepatic HBV-RNA (iHBV-RNA), and covalently closed circular DNA (cccDNA) were quantified at baseline. Additionally, serum markers (HBV-DNA, HBsAg, HBV core-related antigen [HBcrAg] and HBV-RNA) and HBV-specific T cell responses were analysed at baseline and longitudinally throughout follow-up.

**Results:**

After a median follow-up of 34 months, 22/27 patients (82%) remained off-therapy, of whom 8 patients (30% of the total cohort) lost HBsAg. Baseline HBsAg significantly correlated with iHBV-DNA and iHBV-RNA, and these parameters were lower in patients who lost HBsAg. All patients had similar levels of detectable cccDNA regardless of their clinical outcome. Patients achieving functional cure had baseline HBsAg levels ≤1,000 IU/ml. Similarly, an increased frequency of functional HBV-specific CD8+ T cells at baseline was associated with sustained viral control off treatment. These HBV-specific T cell responses persisted, but did not increase, after treatment withdrawal. A similar, but not statistically significant trend, was observed for HBV-specific CD4+ T cell responses.

**Conclusions:**

Decreased cccDNA transcription and low HBsAg levels are associated with HBsAg loss upon NA discontinuation in patients with HBeAg-negative CHB. The presence of functional HBV-specific T cells at baseline are associated with a successful outcome after treatment withdrawal.

**Lay summary:**

Nucleos(t)ide analogue therapy can be discontinued in a high proportion of chronic hepatitis B patients without cirrhosis. The strength of HBV-specific immune T cell responses may contribute to successful viral control after antiviral treatment interruption. Our comprehensive study provides in-depth data on virological and immunological factors than can help guide individualised therapy in patients with chronic hepatitis B.

## Highlights

• Stopping NA is feasible in a high proportion of HBeAg-negative CHB patients.
• Low baseline HBsAg titres identify patients who will achieve functional cure.
• Reduced cccDNA activity is associated with HBsAg loss after NA discontinuation.
• HBV-specific T cell functionality may influence patient outcome upon NA withdrawal.

## Graphical abstract

**Figure:**
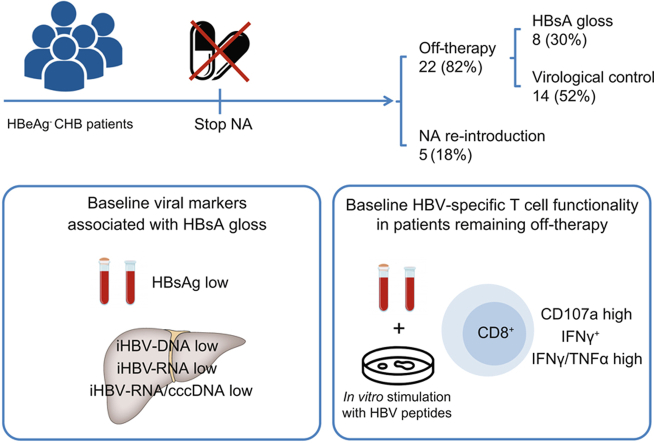

## Introduction

Functional cure, defined as the loss of HBsAg, with or without the development of anti-HBs antibodies, is regarded as the optimal treatment endpoint in patients with HBeAg-negative chronic hepatitis B (CHB). However, despite more than 10 years on nucleos(t)ide analogue (NA) therapy, this endpoint is only achieved in 1–5% of patients.^1^^,^^2^ The latest EASL guidelines propose NA discontinuation in selected HBeAg-negative patients without cirrhosis who have achieved long-term (>3 years) virological suppression on treatment, if close clinical monitoring can be guaranteed after NA discontinuation.^2^ These recommendations are based on data from studies evaluating treatment withdrawal in Caucasian HBeAg-negative patients with complete viral suppression on NA therapy^3^^,^^4^ where the rate of HBsAg loss after NA discontinuation reached 20%. Importantly, a high proportion of these patients achieved sustained virological remission (low HBV-DNA levels and normal alanine aminotransferase [ALT] levels). Despite this recommendation, some clinicians may be reluctant to offer NA withdrawal to their patients owing to the high probability of viral rebound after stopping antiviral therapy (serum HBV-DNA >2,000 IU/ml in about 55–70%).^5^ However, viral relapses are not always associated with ALT flares and, when transitory, may lead to long-term virological remission. In addition, an accurate definition of relapse and criteria for NA re-introduction are needed to prevent premature re-treatment that may impair the possibility of remission or functional cure.^6^

Several studies have suggested that patients with lower circulating HBsAg at the time of NA withdrawal have an increased probability of remaining off-therapy or achieving HBsAg loss^4^^,^^7^ although other studies have failed to find an association.^8^^,^^9^ The evaluation of the prognostic value of other serum markers of HBV replication such as HBV core-related (HBcrAg) and HBV-RNA may be of interest, but data are scarce.^7^^,^^10^^,^^11^ To date, no study has evaluated the significance of HBV replication markers within the liver, such as total intrahepatic HBV-DNA (iHBV-DNA), covalently-closed circular DNA (cccDNA) or intrahepatic HBV-RNA (iHBV-RNA) in relation to NA therapy discontinuation.

As in naturally-resolving HBV infection,^12^ the HBV-specific immune response plays an important role in determining the outcome of viral control after NA discontinuation. A recent study has shown that patients with a higher frequency of HBV-specific T cell responses are less likely to develop ALT flares when stopping NAs.^13^ One study has also shown a tendency for HBV-specific T cell responses to increase after NA cessation, that can be further enhanced by PD-1 blockade.^14^ To our knowledge, this is the first study to evaluate a number of viral and immunological parameters simultaneously. Therefore, our objective was to assess the proportion of patients with HBeAg-negative CHB achieving HBsAg loss or virological control after NA interruption, and to identify potential biomarkers associated with predicting outcome. For this purpose, we longitudinally analysed HBV-specific T cell responses in parallel with peripheral and intrahepatic virological markers after NA discontinuation in a well-characterised cohort of HBeAg-negative CHB patients.

## Materials and methods

### Patients

This is a prospective single-centre study involving 27 patients with HBeAg-negative CHB with complete virological control (undetectable HBV-DNA and normal ALT levels) for at least 3 years under NA therapy (entecavir or tenofovir). Exclusion criteria included advanced liver disease (F3–F4 according to METAVIR by liver biopsy or previous diagnosis of cirrhosis), immunosuppressive therapy, hepatocellular carcinoma, or coinfection with HIV, HCV, or HDV.

The study was approved by the Hospital Clinic institutional Ethics Committee and was conducted in compliance with the principles of the Declaration of Helsinki, Good Clinical Practice guidelines, and local regulatory requirements. All patients provided written informed consent before screening.

### Study design

All patients underwent a liver biopsy before treatment withdrawal (baseline). Serum samples were collected at baseline and at 3, 6, 12, 18, and 24 months during a follow-up period. Peripheral blood mononuclear cells (PBMCs) were also obtained at baseline, 3, and 12 months after NA discontinuation. Assessments after treatment interruption included clinical evaluation, standard laboratory testing for liver function, HBsAg, and HBV-DNA monthly during the first 6 months and then every 3–4 months until the 24-month follow-up.

### Re-introduction criteria and study endpoints

Criteria for re-introduction of antiviral treatment during follow-up were as follows:

• Two consecutive ALT measurements >10 upper limit of normal (ULN) regardless of the HBV-DNA level.
• ALT >5–10 ULN and HBV-DNA >2,000 IU/ml persisting for ≥28 days (4 weeks).
• ALT >2–5 ULN and HBV-DNA >2,000 IU/ml persisting for ≥6 months.
• Need for immunosuppressive treatment.

The primary efficacy endpoint was the proportion of patients achieving HBsAg loss (functional cure) or virological control (patients who remained off-therapy being HBsAg positive) after treatment withdrawal. Secondary endpoints included the analysis of virological and immunological markers as potential predictors of response.

Full materials and methods regarding the assessment of virological parameters, immune responses and statistical analysis are available in the Supplementary data. Further details regarding the materials used can be found in the CTAT table.

## Results

### Patient characteristics and outcome after NA therapy discontinuation

The overall demographic and baseline clinical characteristics of the study cohort are shown in Table 1, with the individual patient data in Table S1. Within the cohort studied, 93% were Caucasian and 78% of patients were male with a median age of 56 years (IQR 45–61). In line with the geographical location of this study, the majority of patients (78%) were infected with HBV genotype D. All patients had previously been treated with NA therapy for a median period of 8 years; with 20 (74%) patients receiving tenofovir and 7 (26%) entecavir. Of note, HBsAg levels remained stable before NA treatment discontinuation in all patients (Supplementary Fig. S1). All patients had normal ALT values at baseline (<40 IU/L), with no patient presenting with advanced fibrosis (F3–F4) according to liver stiffness and histological assessment.

**Table 1:** Characteristics of the entire cohort at baseline and during follow-up according to clinical outcome.

Variables	ALL patients (n = 27)	HBsAg loss (n = 8)	Virological control (n = 14)	Treatment re-introduction (n = 5)	*p* value
Sex (male)	21 (78%)	7 (87%)	10 (71%)	4 (80%)	0.67
Age (years)	56 (45–61)	60 (57–63)	51 (43–56)	46 (44–65)	0.17
Duration of NA therapy (years)	8 (7–13)	13 (9–15)	8 (7–12)	8 (7–11)	0.18
NA therapy					
Tenofovir	20 (74%)	8 (100%)	9 (64%)	3 (60%)	0.13
Entecavir	7 (26%)	0	5 (36%)	2 (40%)	
Baseline elastography (kPa)	4.8 (4.0–5.5)	4.3 (3.3–5.1)	4.8 (3.9–5.3)	4.8 (3.9–5.3)	0.28
Last elastography (kPa; n = 19)	5.3 (4.3–6.1)	4.4 (3.7–5.4)	5.7 (4.2–6.7)	5.3 (4.9–5.7)	0.19
Fibrosis stage					
F0–1	25 (93%)	7 (88%)	13 (93%)	5 (100%)	0.70
F2	2 (7%)	1 (12%)	1 (7%)	0	
HBV genotype					
A	3 (11%)	0	2 (14%)	1 (20%)	0.31
C	1 (4%)	0	1 (7%)	0	
D	21 (78%)	6 (75%)	11 (78%)	4 (80%)	
F	2 (7%)	2 (25%)	0	0	
Baseline ALT (IU/L)	23 (17–26)	21 (17–26)	23 (19–28)	16 (15–27)	0.45
ALT peak ≥2 ULN	17 (63%)	3 (36%)	9 (64%)	5 (100%)	0.07
ALT peak ≥5 ULN	8 (29%)	3 (36%)	2 (14%)	3 (60%)	0.13
ALT peak ≥10 ULN	6 (22%)	3 (36%)	1 (7%)	2 (40%)	0.15
Last ALT (IU/L)	22 (16–30)	17 (16–20)	28 (23–33)	27 (22–36)	0.004
HBV-DNA peak 2,000 IU/ml	21 (78%)	3 (37%)	13 (93%)	5 (100%)	0.005
HBV-DNA peak ≥20,000 IU/ml	12 (44%)	2 (25%)	5 (36%)	5 (100%)	0.019
**Serum virological markers**					
Baseline qHBsAg (IU/ml)	1,310 (556–3,031)	70 (13–507)	2,020 (1,089–4,361)	2,122 (933–3,158)	0.002
≤2,000 IU/ml	17 (63%)	8 (100%)	7 (50%)	2 (40%)	0.03
≤1,000 IU/ml	11 (41%)	7 (87%)	3 (21%)	1 (20%)	0.006
≤100 IU/ml	5 (18%)	5 (63%)	0	0	0.001
Last qHBsAg (IU/ml)	372 (0–1,689)	0	1,368 (175–2,749)	661 (350–1,953)	0.82
≤2,000 IU/ml	21 (28%)	n.a.	9 (64%)	4 (80%)	0.48
≤1,000 IU/ml	17 (63%)	n.a.	6 (43%)	3 (60%)	0.62
≤100 IU/ml	10 (37%)	n.a.	2 (14%)	0	0.53
Delta qHBsAg (Log IU/ml)	-0.22 -(0.58–0.16)	n.a.	-0.22 -(0.66–0.14)	-0.39 -(0.51–0.17)	0.92
Baseline HBcrAg (positive)	13 (48%)	2 (25%)	8 (57%)	3 (60%)	0.29
Baseline HBcrAg (log U/ml)∗	3.2 (2.8–5.1)	3.9 (2.8–5.1)	3.0 (2.8–3.3)	3.0 (2.8–3.4)	0.97
Last HBcrAg (positive)	8 (29%)	1 (12%)	5 (36%)	2 (40%)	0.53
Baseline HBV-RNA (positive)	11 (41%)	1 (12%)	7 (50%)	3 (60%)	0.14
Baseline HBV-RNA (copies/ml)∗	136 (96–842)	n.a.	101 (96–477)	840 (603–922)	0.07
Last HBV-RNA (positive)	10 (37%)	0	7 (50%)	3 (60%)	0.53
**Liver virological markers at baseline**					
iHBV-DNA (copies/cell)	0.36 (0.14–1.02)	0.04 (0.02–0.13)	0.58 (0.25–1.15)	0.91 (0.53–1.33)	0.001
cccDNA (copies/cell)	0.09 (0.035–0.36)	0.04 (0.01–0.40)	0.11 (0.06–0.36)	0.05 (0.02–0.29)	0.32
iHBV-RNA (positive)†	12/18 (67%)	2/7 (14%)	7/8 (87%)	3/3 (100%)	0.009
iHBV-RNA (copies/25 ng total RNA)∗^,^†	203 (37–4,417)	n.a.	655 (28–4,417)	123 (29–184)	0.15
iHBV-RNA/cccDNA∗^,^†	3,834 (76–34,780)	n.a.	5,642 (76–34,780)	3,835 (3,174–28,996)	0.72

Categorical and quantitative data are expressed as n (%) and median (IQR), respectively. Groups were compared using the Chi-square test for categorical variables and the ANOVA test for continuous variables.

ALT, alanine aminotransferase; cccDNA, covalently closed circular DNA; iHBV-DNA, intrahepatic HBV-DNA; iHBV-RNA, intrahepatic HBV-RNA; n.a., not applicable; ULN, upper limit of normal.

∗ Analysis of median (IQR) values for HBcrAg, HBV-RNA, iHBV-RNA, and iHBV-RNA/cccDNA was performed only considering patients with positive results.

† Data available for 18 patients. One patient in the HBsAg loss group had detectable iHBV-RNA <LLQ.

**Fig. 1:**
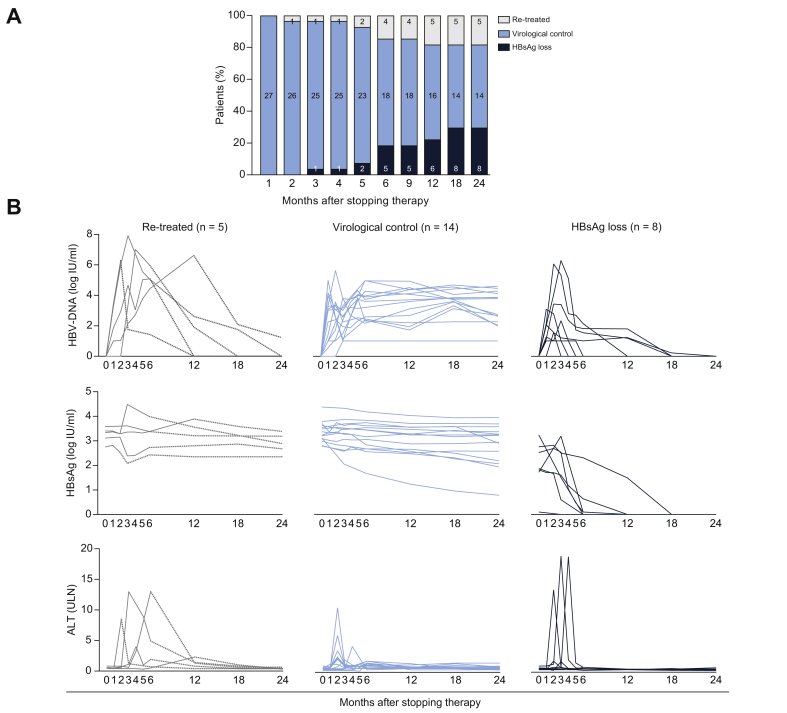
Patient outcome and viral kinetics after NA discontinuation. (A) Patient outcome after NA discontinuation. Bars represent the percentage of patients with HBsAg loss, virological control, or re-treatment. The number of patients is shown within the bars. (B) HBV-DNA, HBsAg, and ALT kinetics. For re-treated patients, the dotted line represents the evolution after treatment re-introduction. ALT, alanine aminotransferase; NA, nucleos(t)ide analogue.

After a median follow-up of 34 (26–37) months, 22 patients (82% of the total cohort) remained off-therapy, with 8 individuals (representing 30% of the total cohort) achieving functional cure, as defined by the loss of HBsAg (Fig. 1A and Table 1). Notably, the median time to HBsAg clearance in these patients was 25 (22–33) weeks, with 5 of these 8 patients developing detectable anti-HBs antibody. At the end of the follow-up period, among those patients who remained off-therapy but were positive for HBsAg (n = 14, 52%), considered ‘virological controllers’, 5 met the criteria for HBeAg-negative chronic infection with normal ALT values and HBV-DNA <2,000 IU/ml. The remaining patients were in a ‘grey zone’; 7 had HBV-DNA levels >2,000 IU/ml with normal ALT values, with a further 2 patients presenting with elevated ALT levels that remained <2 ULN. Of the 27 CHB patients, only 5 patients (18%) required NA re-introduction after stopping NAs (Fig. 1A). The reasons for treatment re-introduction were: early flare in HBV-DNA and persistent ALT levels >10 ULN (n = 1), high viraemia with persistent 2–5 ULN ALT levels for at least 4 weeks (n = 3), and the need for high dose corticosteroid therapy for more than 7 days (n = 1, who also had an ALT >2 ULN and HBV-DNA >20,000 IU/ml >8 weeks). We did not find any difference according to outcome after NA discontinuation when considering patient characteristics including sex, age, type, and duration of antiviral therapy or HBV genotype (Table 1 and Supplementary Table S1).

### HBV-DNA, ALT, and HBsAg kinetics after NA withdrawal

In this cohort, HBV-DNA viraemia peaked within 1–3 months of stopping NA therapy (Fig. 1B). Most patients had a virological relapse defined as either an increase in HBV-DNA above 2,000 IU/mL (78%) or 20,000 IU/mL (44%).

ALT flares were observed in some patients during the ‘off-therapy’ period, although these were mostly mild (Table 1). Patients with ALT flare >10 ULN presented with diverse clinical outcomes with only 2 out of 6 patients needing treatment re-introduction, whereas 4 out of 6 remained off-therapy, including 3 who achieved HBsAg loss (Table 1 and Fig. 1B). Neither HBV-DNA nor ALT kinetics predicted the clinical evolution during follow-up. The overall reduction in HBsAg levels was -0.22 -(0.58–0.16) log IU/ml during follow-up with no differences noted between virological controllers and patients re-starting therapy (Fig. 1B). The evolution of the remaining markers (HBcrAg and serum HBV-RNA) is shown in Table 1 and Supplementary Table S1.

### Baseline HBsAg significantly correlates with iHBV-DNA and iHBV-RNA, but not cccDNA

Serum and liver parameters of HBV replication were analysed before treatment interruption (Table 1 and Supplementary Table S1). The median baseline HBsAg titre in our cohort was 1,310 (556–3031) IU/ml, with 11 patients (41%) ≤1.000 IU/ml and 5 (18%) ≤100 IU/ml. Despite long-term NA therapy, all patients had detectable iHBV-DNA and cccDNA at baseline. In contrast, only 11 (41%) and 13 (48%) patients had detectable serum levels of HBV-RNA and HBcrAg, respectively. Intrahepatic HBV-RNA was detected in 67% of liver samples at baseline. Notably in all patients with undetectable iHBV-RNA (n = 6), we were also unable to detect HBV-RNA in their matched serum samples. In 8 patients we detected both iHBV-RNA and serum HBV-RNA, whereas in 4 patients only iHBV-RNA was detectable. In addition, 5 out of 7 patients with virological control and 2 out of 3 patients needing re-treatment had detectable iHBV-RNA and positive serum HBV-RNA levels.

**Fig. 2:**
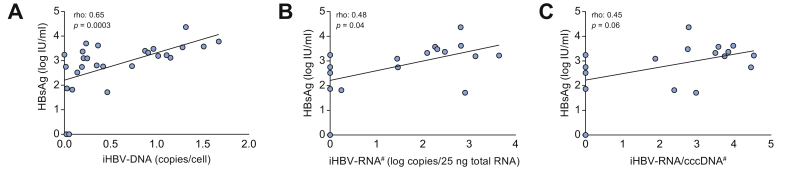
Correlations between HBsAg and intrahepatic replication markers before NA discontinuation. Correlation between HBsAg and (A) iHBV-DNA, (B) iHBV-RNA, and (C) iHBV-RNA/cccDNA ratio as a measure of cccDNA transcriptional activity. Rho, Spearman’s correlation coefficient. ^#^Data available for 18 patients. cccDNA, covalently closed circular DNA; iHBV-DNA, intrahepatic HBV-DNA; iHBV-RNA, intrahepatic HBV-RNA; NA, nucleos(t)ide analogue.

We analysed the correlations between baseline serum and intrahepatic viral markers (Fig. 2 and Supplementary Table S2). HBsAg correlated with both iHBV-DNA (rho = 0.65, *p* = 0.0003) and iHBV-RNA (rho = 0.48, *p* = 0.04), but not with cccDNA. Furthermore, serum HBV-RNA correlated with iHBV-DNA and iHBV-RNA (rho = 0.50, *p* = 0.008 and rho = 0.56, *p* = 0.01, respectively). We did not observe any correlation between the duration of prior NA therapy and the expression of any virological parameter tested (data not shown).

### Low baseline HBsAg levels are associated with HBsAg loss upon NA withdrawal

**Fig. 3:**
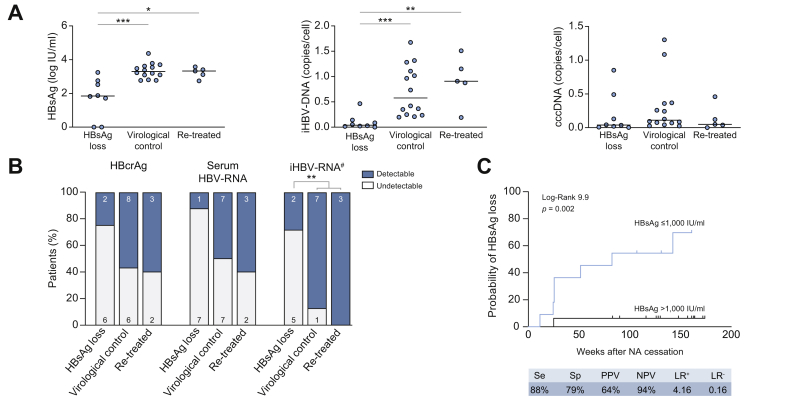
Association between baseline virological markers and outcome after NA discontinuation. (A) Baseline HBsAg, iHBV-DNA, and cccDNA levels according to patient outcome. The red lines indicate median values. (B) Proportion of patients with detectable HBcrAg, serum HBV-RNA, and iHBV-RNA according to outcome. Mann-Whitney (A) and Chi-squared tests (B) were used for comparison, ∗*p* <0.05, ∗∗*p* <0.01, ∗∗∗*p* <0.001. (C) Kaplan-Meier curves for HBsAg loss after NA interruption according to HBsAg levels at baseline. ^#^Data available for 18 patients. cccDNA, covalently closed circular DNA; iHBV-DNA, intrahepatic HBV-DNA; iHBV-RNA, intrahepatic HBV-RNA; LR, likelihood ratio; NA, nucleos(t)ide analogue; NPV, negative predictive value; PPV, positive predictive value; Se, sensibility; Sp, specificity.

Patients achieving HBsAg loss had lower HBsAg and iHBV-DNA but similar cccDNA levels at baseline, compared with the individuals sustaining virological control and those requiring re-treatment (Table 1 and Fig. 3A). Importantly, 5 out of 7 (71%) patients who achieved HBsAg loss had undetectable iHBV-RNA at baseline (*p* <0.01), whereas 91% of patients who remained HBsAg-positive had detectable levels of iHBV-RNA (Table 1 and Fig. 3B). In addition, baseline HBcrAg and serum HBV-RNA were more frequently undetectable in patients who achieved HBsAg loss than in patients who did not (HBcrAg: 75% *vs*. 42%; *p* = 0.12 and HBV-RNA: 88% *vs.* 47%; *p* = 0.053), although the differences did not reach statistical significance. As shown in Fig. 3C, the probability of HBsAg loss was higher among patients with low HBsAg ≤1,000 IU/ml (log rank 9.9, *p* = 0.002). Combining HBsAg levels ≤1,000 IU/ml and undetectable serum HBV-RNA did not improve the predictive value for HBsAg loss (log rank 6.7, *p* = 0.009; Supplementary Fig. S2). Although virological parameters predicted HBsAg loss, we did not see differences between the virological control and relapse groups.

### HBV-specific T cell responses at baseline are associated with virological control off-treatment

**Fig. 4:**
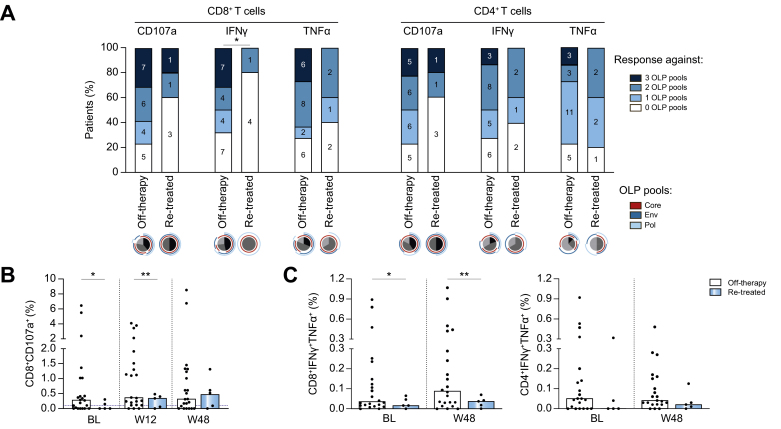
HBV-specific T cell responses at baseline and during follow-up. (A) Percentage of patients with baseline HBV-specific T cell responses (≥0.1% positive cells after subtracting the unstimulated control) and hierarchy of the T cell responses according to the different overlapping peptide (OLP) pools. (B) Degranulating CD8+ T cells after *in vitro* expansion with core OLP. (C) Polyfunctional T cells co-expressing IFNγ and TNFα after core OLP stimulation at baseline and 48 weeks after treatment withdrawal. Bars represent the median and dotted lines the 0.1% cut-off for positive T cell responses minus the unstimulated control (B and C). Chi-square test (A) and Welch’s *t* test (B, C) were used to compare groups, ∗*p* <0.05, ∗∗*p* <0.01.

To evaluate the impact of the HBV-specific T cell response on the clinical outcome after NA discontinuation, we investigated the effector capacity of HBV-specific CD4+ and CD8+ T cells after *in vitro* stimulation with overlapping peptides (OLP) spanning the core, envelope, or polymerase proteins. A representative gating strategy and example flow cytometric plots are shown in Supplementary Fig. S3 and individual data showing CD8+ and CD4+ T cell responses at all time points are shown in Supplementary Fig. S4. Interestingly, an increased proportion of patients that remained off-therapy had functional HBV-specific CD8+ T cell responses against epitopes from more than one HBV protein before NA withdrawal compared to those requiring NA re-introduction (68% *vs.* 20% *p* = 0.048 for IFNγ production and 77% *vs.* 40% *p* = 0.099 for CD107a expression, respectively. Of note, we defined an HBV-specific T cell response as ≥0.1% after subtraction of the unstimulated control; Fig. 4A). Interestingly, for both CD8+ and CD4+ T cells, responding cells to all viral antigens were more likely to be detectable at baseline in patients who remained off-therapy than in the re-treatment group. Indeed, CD4+ and CD8+ T cells producing IFNγ and TNFα in response to any peptide pool were absent in all patients that had NA re-introduction. However, no statistically significant differences were observed within groups likely attributable to the number of patients falling in the re-treatment group.

The percentage of degranulating CD8+ T cells (CD107a) in response to stimulation with HBV-core OLP was significantly higher at baseline and after 12 weeks of stopping NA treatment in patients remaining off-therapy than in those requiring NA re-introduction (*p* = 0.039 and 0.0093, respectively; Fig. 4B). However, these differences in CD107a responses between patient groups were not maintained when focusing on OLP encompassing the envelope and polymerase proteins (Supplementary Fig. S5).

When we investigated the presence of polyfunctional CD8+ and CD4+ T cells (cells co-expressing IFNγ and TNFα), we observed that the percentage of core-specific CD8+ T cells producing IFNγ/TNFα at baseline was increased among patients who remained off-therapy compared with those patients needing treatment re-introduction (*p* = 0.031; Fig. 4C). Importantly, this increase persisted for more than a year of follow-up whilst off-therapy (*p* = 0.01). A similar trend was observed when probing effector CD4+ T cells co-producing IFNγ/TNFα, however, the differences between outcome groups did not reach statistical significance (Fig. 4C). Likewise, no differences were observed between groups of patients (off-therapy and treatment re-introduction) regarding the presence of polyfunctional T cell responses against envelope and polymerase proteins (Supplementary Fig. S6).

We also evaluated if there was any association between T cell responses and the development of clinically relevant ALT flares during follow-up. As shown in Supplementary Fig. S7, we observed that HBV-specific CD8+ and CD4+ T cell responses at baseline were similar between patients regardless of whether they developed an ALT flare (using a threshold of ≥2 ULN).

### HBV-specific responses do not increase following treatment withdrawal

**Fig. 5:**
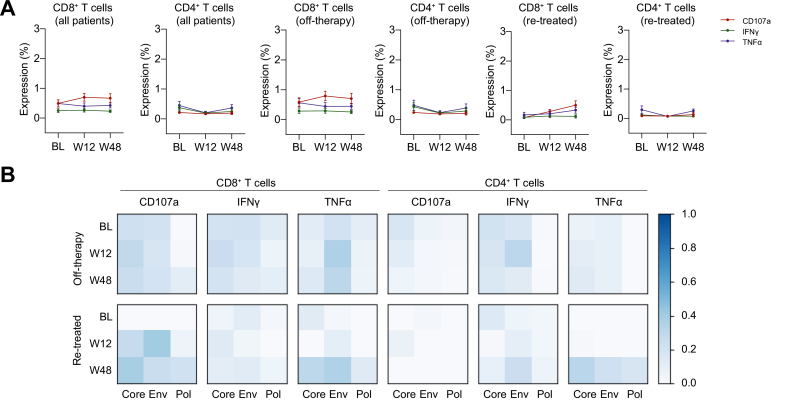
HBV-specific T cell responses do not increase after NA withdrawal. (A) Longitudinal analysis of HBV-specific T cell responses after stopping therapy (entire cohort and broken down by clinical outcome: off-therapy and re-treated). Data are expressed as mean ± SEM. (B) Heat map showing normalised median frequencies of HBV-specific T cell responses against each OLP at baseline (BL), week 12 (W12) and week 48 (W48) after treatment discontinuation. Normalisation was performed by scaling maximum and minimum values per marker between 0 and 1. Absence of ∗ indicates no differences compared to baseline in each group (Wilcoxon Signed-Rank test). Env, envelope; NA, nucleos(t)ide analogue; Pol, polymerase.

To investigate the impact of NA discontinuation on functional HBV-specific T cells, we performed a longitudinal analysis of the T cell responses against HBV OLP at 12 and 48 weeks after NA withdrawal. We found that the frequency of either HBV-specific CD8+ or CD4+ T cells detectable by intracellular cytokine staining for the antiviral mediators CD107a, IFNγ, and/or TNFα did not significantly change during follow-up compared to baseline (Fig. 5A and Supplementary Fig. S4). Similarly, when classifying patients according to whether they remained off-therapy or required treatment re-introduction, no significant changes in any effector functions to any of the peptide pools were detected at week 12 or 48 compared with baseline (Fig. 5B).

**Fig. 6:**
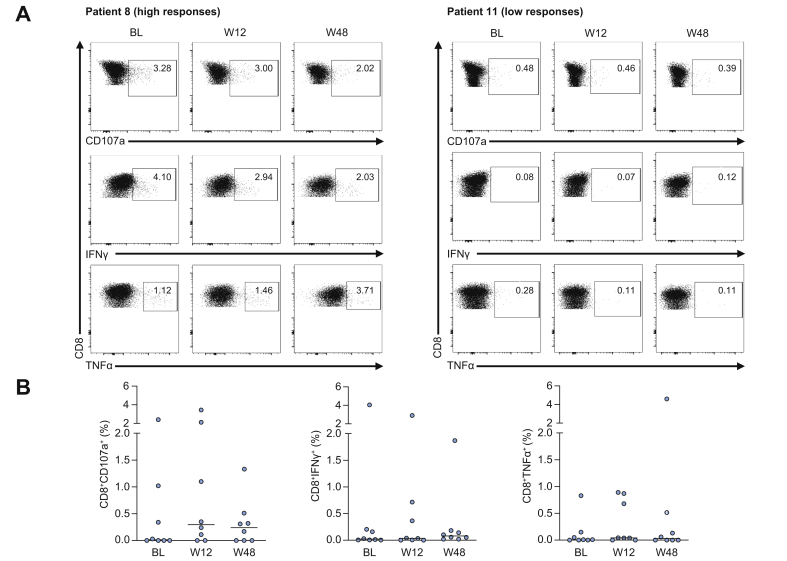
HBV core-specific CD8+ T cell responses in patients who achieved HBsAg loss after treatment withdrawal. (A) Representative dot plots of HBV-specific effector CD8+ T cells from 2 patients who lost HBsAg. (B) Longitudinal HBV-specific T cell responses against core OLP in patients that achieved HBsAg loss after stopping therapy. No differences were observed at weeks 12 and 48 when comparing to baseline (Wilcoxon Signed-Rank test).

As HBsAg loss with or without the production of anti-HBs represents the best predictor of success upon discontinuation of NA therapy, we analysed HBV-specific CD8+ and CD4+ T cell responses within this small group of individuals. Notably, the effector CD8+ T cell response to antigenic stimulation in these patients was heterogeneous, with a significantly greater magnitude of response to HBV OLP stimulation in some patients, and with absent or very weak responses for all mediators (CD107a, IFNγ or TNFα) in others (Fig. 6A). The longitudinal analysis after NA cessation did not show significant changes in the response rates in this subgroup of patients (Fig. 6B). Similar results were obtained for HBV-specific CD4+ T cell responses (data not shown).

### The strength of HBV-specific T cell responses does not correlate with serum or intrahepatic virological markers

We next explored whether differences in the HBV-specific T cell response were associated with serum or intrahepatic virological parameters. As we noted, baseline HBsAg levels ≤1,000 IU/ml were predictive of HBsAg clearance in our cohort, we classified patients accordingly and analysed their HBV-specific CD8+ and CD4+ T cell responses at baseline. The proportion of patients with HBV-specific effector T cells as well as the frequency of polyfunctional T cells co-producing IFNγ/TNFα were similar regardless of HBsAg titres (Supplementary Fig. S8). We did not find any significant correlation between peripheral HBV-specific T cell responses and other serum (*e.g.* HBcrAg) or liver replication markers (iHBV-DNA, cccDNA, iHBV-RNA, or iHBV-RNA/cccDNA ratio).

### Safety

Importantly, no patient developed hepatic decompensation, hepatocellular carcinoma, or died during follow-up. Bilirubin levels greater than 2 mg/dl or prolongation of prothrombin time were not detected at any time point, even amongst those individuals requiring treatment re-introduction. No other adverse events were detected during the follow-up period. All patients requiring NA re-introduction had a subsequent good response with entecavir or tenofovir, returning to undetectable serum HBV-DNA and a normal ALT within 6 months. Furthermore, we observed no change in liver stiffness assessment at the 24-month follow-up in 19 of our 27 patients compared with their matched baseline assessment or when comparing treatment outcome groups (Table 1).

## Discussion

Despite lifelong treatment with NA being safe and effective in patients with HBeAg-negative CHB, the overall rate of HBsAg loss remains low.^1^^,^^2^ Several new direct-acting antivirals and immunomodulatory compounds are in clinical evaluation with the aim of achieving functional cure of CHB with finite treatment duration.^15^ Alternatively, NA discontinuation has been proposed as a promising therapeutic strategy to promote HBsAg loss or at least sustained off-treatment viral control in a considerable proportion of patients.[2], [3], [4], [5]^,^^16^ However, the reported rates of HBsAg loss in NA withdrawal studies have been enormously variable, in part owing to the heterogeneity of patient cohorts, and the absence of standardised treatment re-introduction criteria. To investigate the correlates of outcomes upon NA cessation, we analysed liver and serum markers of HBV replication in parallel with assessment of HBV-specific T cell immunity in a well-characterised cohort of patients with HBeAg-negative CHB undergoing NA discontinuation.

In our study, after a median follow-up of 2.8 years from treatment interruption, 82% of patients remain off-therapy and 8 (30% of the total cohort) continued to remain HBsAg negative. Virological relapses were observed in the majority (78%) of patients although these were not always associated with biochemical relapses. Indeed, most of the ALT flares were mild and temporary, leading to long-term virological and biochemical remission with no patients developing liver dysfunction. Our results agree with those reported by Berg *et al.*,^3^ the only randomised trial comparing the strategy of NA-therapy maintenance and therapy withdrawal, which included a comparable population in terms of genotypic and clinical characteristics.

Although treatment re-introduction criteria differ among the current published studies, we and others have shown that lower HBsAg titres at the time of NA withdrawal are associated with an increased probability of HBsAg loss.^3^^,^^4^^,^^6^^,^[17], [18], [19] In our study, a threshold value for HBsAg of ≤1,000 IU/ml was a good predictor whether a patient went on to lose HBsAg. Likewise, patients who achieved HBsAg loss had lower iHBV-DNA levels at baseline; this marker includes cccDNA, relaxed circular DNA, and integrated HBV-DNA. Indeed, integration of HBV-DNA into the host genome and its contribution to the formation of HBsAg particles^20^ may explain the good correlation between iHBV-DNA and HBsAg in our cohort of HBeAg-negative patients chronically suppressed by NA therapy.

We did not find an association between baseline cccDNA levels and virological outcome. However, most patients achieving HBsAg loss had undetectable iHBV-RNA (71%) and, hence, reduced cccDNA transcriptional activity (iHBV-RNA/cccDNA ratio). This would explain, at least in part, a more favourable outcome in these patients. Indeed, 10 out of 11 patients not achieving HBsAg loss had detectable iHBV-RNA suggesting transcriptionally active cccDNA and a higher risk of viral rebound. We also studied the prognostic value of other surrogate serum markers of cccDNA transcriptional activity such as HBcrAg and serum HBV-RNA.^7^^,^^21^^,^^22^ The proportion of patients with undetectable serum HBV-RNA or HBcrAg at baseline in our cohort was higher among patients achieving HBsAg loss (7/8 and 6/8 patients, respectively), however, these markers did not improve the ability of HBsAg levels to predict functional cure. Importantly, serum HBV-RNA and HBcrAg may have become undetectable in HBeAg-negative patients under long-term NA therapy thus limiting their predictive power.^7^^,^^21^

As adaptive immune responses are important for the control of HBV infection,^12^^,^^23^^,^^24^ we analysed the effector functionality of HBV-specific CD4+ and CD8+ T cells at the time of treatment withdrawal (baseline) and throughout NA discontinuation. At baseline, the proportion of patients with HBV-specific CD8+ T cells expressing CD107a and IFNγ after *in vitro* peptide stimulation was higher amongst patients who subsequently remained off-therapy compared with those that needed treatment re-introduction. The presence of polyfunctional T cells has been associated with an improved control of viral replication.^23^^,^^25^ Accordingly, we found that the frequency of HBV-specific T cell co-producing IFNγ/TNFα were higher both at baseline and 1 year after stopping therapy in patients who remained off-therapy. However, in contrast to recent findings,^14^ treatment withdrawal did not induce any significant change in the HBV-specific CD8+ and CD4+ T cell response after 12 or 48 weeks of follow-up.

Interestingly, patients achieving HBsAg loss presented a heterogeneous HBV-specific effector T cell response both at baseline and during follow-up, with no clear pattern differentiating those patients achieving HBsAg loss after an ALT flare or not. In line with the concept that HBV-specific CD8+ T cells can exert viral control without liver damage,^24^ a recent study showed higher baseline frequencies of core and polymerase-specific CD8+ T cells were predictive of viral control in the absence of a flare after treatment interruption.^13^ Our findings similarly show that higher baseline HBV-specific CD8+ T cells were predictive of virological control off-treatment. In both studies, ALT flares were not associated with significant increases in HBV-specific CD8+ T cell frequency (in the Rivino *et al.*^13^ study they were lower in the flaring group), raising the possibility of alternative immune mechanisms driving liver damage.

Finally, our study also has some limitations. First, although an HBsAg threshold was not an inclusion criterion, our cohort is characterised by a proportion of patients with low HBsAg levels at baseline which might have influenced the improved success rates in this study. Nonetheless, when excluding the 2 patients with lower qHBsAg (<1 IU/ml), the statistical analysis regarding baseline differences and predictive factors remained the same. Second, because of the limited amount of liver tissue, we were not able to assess HBV-DNA integration or to study innate immune responses within the liver,^26^ or the contribution of liver-resident T cells to viral control.[27], [28], [29] Lastly, we cannot rule out future transition to a different HBV infection phase in patients with virological control, thus these patients remain under clinical monitoring.

In summary, stopping NA therapy is feasible in a high proportion of HBeAg-negative patients without cirrhosis. Although lower cccDNA transcriptional activity after long-term antiviral therapy is associated with successful outcome after NA withdrawal, intrahepatic viral parameters do not seem to improve the predictive capacity of readily available serum markers such as HBsAg. Indeed, low HBsAg levels (≤1,000 IU/ml) may offer predictive value in assessing those patients in whom no specific intervention other than stopping antiviral therapy and close monitoring would be a valid therapeutic strategy for achieving functional cure. For those patients with a less favourable virological profile, the strength of HBV-specific T cell responses at baseline may contribute to the outcome of treatment interruption. The latter group may be more responsive to novel finite immunotherapeutic options after NA discontinuation to increase the rate of HBsAg loss.

### Abbreviations

ALT, alanine aminotransferase; BL, baseline; cccDNA, covalently closed circular DNA; CHB, chronic hepatitis B; Env, envelope; HBcrAg, HBV core-related antigen; iHBV-DNA, intrahepatic HBV-DNA; iHBV-RNA, intrahepatic HBV-RNA; LR, likelihood ratio; NPV, negative predictive value; NA, nucleos(t)ide analogue; OLP, overlapping peptides; PBMC, peripheral blood mononuclear cells; Pol, polymerase; PPV, positive predictive value; rho, Spearman’s correlation coefficient; Se, sensibility; Sp, specificity; ULN, upper limit of normal; W12, week 12; W48, week 48.

## Financial support

This study was sponsored by the 10.13039/501100004587Instituto de Salud Carlos III (ISCIII) through the Plan Estatal de Investigación Científica y Técnica y de Innovación 2013–2016, grants PI16/00111 (SPP), PI15/00151 and PI18/00079 (XF), and PI18/01436 (FRF), co-funded by the 10.13039/501100008530European Regional Development Fund (ERDF), by the Gilead Fellowship Programme, grant GLD15/00274 (XF) and by Plan Estratégico Nacional contra la hepatitis C (Spanish Health Ministry). XF has received support from Secretaria d’Universitats i Recerca del Departament d’Economia i Coneixement (grant 2017_SGR_1753) and CERCA Programme/10.13039/501100002809Generalitat de Catalunya. SL has received grants from 10.13039/100008318Asociación Española para el Estudio del Hígado (AEEH) and Societat Catalana de Digestologia (SCD). GK is supported by Fundación IBEROSTAR. MGL is supported by the i-PFIS program (fellowship IFI18/00006) and SRT by the Rio Hortega program (fellowship CM17/00015) of the 10.13039/501100004587ISCIII, both co-funded by the 10.13039/501100004895European Social Fund (ESF). SRT is supported by an initiation research grant from SCD and the Emili Letang end-of-residency prize from Hospital Clínic de Barcelona. MKM and LJP are supported by Wellcome Trust Investigator award 214191/Z/18/Z and CRUK Immunology grant 26603. This work was performed within the framework of the RHU CirB-RNA (ANR-17-RHUS-0003) that is part of the second programme ‘Investissements d’Avenir’ operated by the French National Research Agency (ANR) (FZ and BT).

## Authors’ contributions

Contributed to the study concept and design: XF, SL, SPP. Patient selection/inclusion: SL, SRT, ZM, XF. Acquired data: MGL, SL, SRT, EGP, EP, TL, GK, CB, FRF, ZM, XF, SPP. Contributed to analysis and interpretation of data: MGL, SL, XF, SPP, JJL, FZ, BT, LJP, MKM. Drafted the manuscript: SL, MGL, SPP. Contributed to critical revisions and approved the final manuscript: all authors.

## Data availability

Data regarding patients included in the study are confidential. Additional information relevant to readers is provided as Supplementary data.

## Conflicts of interest

SL has received lecture and advisory fees from Gilead, Abbvie, and MSD. XF has acted as advisor for Gilead and Abbvie. SRT has received lecture fees from Gilead and Abbvie. ZM has received fees for lectures and consulting from Gilead and Abbvie, and a research grant from Gilead. The Maini lab has received research funding from Gilead, Hoffmann La Roche and Immunocore. MKM has sat on advisory boards/provided consultancy for Gilead, Hoffmann La Roche, Immunocore, VIR, Galapagos NV, GSK, Abbvie, and Freeline. LJP has received fees from Gilead for consultancy work. FZ received research grants and consultancy honoraria from Hoffmann-La-Roche, Gilead Sciences, Janssen, Assembly Biosciences, Arbutus, Contravir, Sanofi, and Transgene. All other authors have declared no conflicts of interest.

Please refer to the accompanying ICMJE disclosure forms for further details.
