# Supplementary material for: Viral and immune factors associated with successful treatment withdrawal in HBeAg-negative chronic hepatitis B patients
Source: J Hepatol. 2021 May;74(5):1064–74. doi: 10.1016/j.jhep.2020.11.043 (PMC8062913; doi:10.1016/j.jhep.2020.11.043)
Supplement: Multimedia component 1 [file mmc1.docx]

**Serum and intrahepatic viral markers and HBV-specific T cell responses in HBeAg-negative chronic hepatitis B patients undergoing therapy discontinuation**

Mireia García-López, Sabela Lens, Laura J. Pallett, Barbara Testoni, Sergio Rodríguez-Tajes, Zoe Mariño, Concepción Bartres, Ester García-Pras, Thais Leonel, Elena Perpiñán, Juan José Lozano, Francisco Rodríguez-Frías, Georgios Koutsoudakis, Fabien Zoulim, Mala K. Maini, Xavier Forns, Sofía Pérez-del-Pulgar.

**Table of Contents**

[Supplementary materials and methods 2](#_Toc50118535)

[Table S1 7](#_Toc50118536)

[Table S2 8](#_Toc50118537)

[Table S3 9](#_Toc50118538)

[Table S4 9](#_Toc50118539)

[Fig. S1 10](#_Toc50118540)

[Fig. S2 10](#_Toc50118541)

[Fig. S3 11](#_Toc50118542)

[Fig. S4 12](#_Toc50118543)

[Fig. S5 1](#_Toc50118544)4

[Fig. S6 15](#_Toc50118545)

[Fig. S7 16](#_Toc50118546)

[Fig. S8 17](#_Toc50118547)

[References 18](#_Toc50118548)

Supplementary materials and methods

***Viral serum parameters***

Standard laboratory tests (upper limit normal for ALT= 40 IU/L) as well as determinations of HBeAg status and anti-HBe were performed by immunoassay using the Advia Centaur® System (Siemens, Erlangen, Germany). Serum HBV-DNA was determined by real-time PCR using the cobas® 6800 system (Roche Diagnostics, Manheim, Germany; LLQ <10 IU/ml). HBsAg was quantified using the ARCHITECT® HBsAg assay (Abbott Laboratories, Chicago, IL, USA; LLQ <0.13 IU/ml). HBcrAg levels were assessed by chemiluminescent enzyme immunoassay using LUMIPULSE® G1200 Analyzer (Fujirebio Europe, Gent, Belgium; LLQ <2.8 log U/ml) according to the manufacturer’s instructions.

Serum HBV-RNA was purified using the QIAamp Viral RNA Mini kit (Qiagen), followed by DNase treatment at 25ºC for 15 min (RNase-Free DNase set, Qiagen). To monitor proper nucleic acid extraction and amplification, a fixed dose of an internal control was added at each sample (Internal RNA extraction control kit, Primer Design, Camberley, UK). 3.5 kb HBV-RNA (pregenomic RNA and precore mRNA) was assessed by real-time RT-PCR using specific primers and Taqman probe detailed in **Table S3**^1^ and the TaqMan® Fast Virus 1-Step Master Mix (Applied Biosystems, Thermo Fisher Scientific, Waltham, MA, USA). Absolute quantification was performed using serial dilutions of an *in vitro* transcribed plasmid containing 1.8 kb of the HBV genome (including the target region for the 3.5 kb HBV-RNA detection) under the control of T7 promoter. We used Probit analysis to determine that the limit of quantification was 6 copies per reaction with 95% probability.

The HBV genotype was determined by direct sequencing and phylogenetic analysis of a fragment of 1137 bp of the viral polymerase (nucleotides 108 to 1244, GenBank accession number NC003977) as described by Tong et al.^2^ Multiple alignments were performed with ClustalW^3^ and maximum likelihood trees were obtained with MEGA X software.^4^

***Virological tissue markers***

All patients underwent a liver biopsy prior to treatment withdrawal (baseline). This biopsy was divided into two parts, one for conventional histological analysis to rule out the presence of advanced fibrosis and the other part was stored in Allprotect Tissue Reagent (Qiagen, Hilden, Germany) at -80°C. Total nucleic acids were extracted from frozen human liver needle biopsies using the MasterPure™ DNA and RNA purification kit (Epicentre, Illumina, San Diego, CA, USA). Nucleic acid preparations were divided into two parts and treated with RNase A or DNase for further total DNA and RNA purification, respectively. The quantity of purified DNA and RNA was measured by NanoDrop Spectrophotometer (NanoDrop Technologies, Wilmington, DE, USA) and Qubit® 3.0 Fluorometer (Invitrogen, Thermo Fisher Scientific). Total intrahepatic HBV-DNA (iHBV-DNA) and cccDNA were determined by real-time PCR, according to the protocol described by Allweiss et al., ^5^ with some modifications. Quantification was performed using the ViiA 7 Real-Time PCR System and primers and TaqMan probe shown in **Table S3**.^1^ For cccDNA determination, purified DNA was pretreated with Plasmid-Safe^TM^ DNase (Epicentre, Lucigen Corporation, Middleton, WI, USA) at 37ºC for 4 h. The standard curve was prepared from serial dilutions of a plasmid containing an HBV monomer (pBR322-HBV). Probit analysis was used to determine that the limit of quantification with 95% probability was 6 copies per reaction for iHBV-DNA and 17 copies per reaction for cccDNA. Viral genome copies were normalized vs the number of cells, estimated by the human β-globin gene copy number (ID Hs00758889_s1, ABI, Thermo Fisher). For intrahepatic 3.5 kb HBV-RNA (iHBV-RNA), we followed the same protocol as described above for serum HBV-RNA.

***Immunological analysis***

PBMCs were isolated by density centrifugation using Ficoll Histopaque (Sigma-Aldrich, St. Louis, MO, USA) and cryopreserved. PBMCs were suspended in RPMI 1640 medium (Thermo Fisher Scientific) supplemented with 10% heat-inactivated human serum (Sigma-Aldrich, St. Louis, MO, USA), 100 U/ml penicillin, 100 ug/ml streptomycin, non-essential and essential amino acids (Thermo Fisher Scientific) and 20 IU/ml rhIL-2 (Miltenyi Biotec, Bergisch Gladbach, Germany). To assess the functionality of HBV-specific T cells, PBMC were seeded at a density of 10^6^ cells/ml and stimulated for 7 days with 1 μg/ml pangenotypic (genotypes A-D) overlapping peptides (OLP) spanning HBV core or envelope proteins (kindly provided by Gilead Sciences, Foster City, CA, USA) or with genotype D polymerase-specific OLP (Peptide Synthesis Core Facility, UPF, Barcelona, Spain). Pangenotypic core and envelope pools consisted of 94 and 166 15-mer peptides, respectively, overlapping by 12 amino acids. The genotype D polymerase pool consisted of 165 15-mer peptides overlapping by 10 amino acids. The final DMSO concentration during *in vitro* expansion was 0.5%.

To exclude the possibility that some polymerase-specific T cell responses were missed because of the use of a genotype D polymerase pool, we compared the presence or absence of polymerase-specific responses between genotype D and non-genotype D patients. Importantly, no statistically significant differences were found between the 2 groups: 5 of 6 (83%) non-genotype D patients and 15 of 21 (71%) genotype D patients showed polymerase-specific T cell responses (Chi-square test, p= 0.56).

Throughout the *in vitro* expansion, cell culture medium was replaced every 3 days. For the final 16 h, cells were restimulated with the respective HBV OLP in the presence of 1 µg/ml brefeldin A (Sigma-Aldrich), 2 µM monensin (Biolegend, San Diego, CA, USA) and an anti-CD107a monoclonal antibody (mAb; Biolegend). To help with gating and as positive control of cytokine production, cells were stimulated with either 2 ng/ml PMA and 1 µg/ml ionomycin (both from Sigma-Aldrich) or 1 µg/ml ProMix™ CEF Peptide Pool (MHC class I-restricted viral peptides from human CMV, EBV and influenza virus; Proimmune Ltd., Oxford, UK). Of each sample at each time point, we included an unstimulated sample as control. After restimulation, cells were washed and stained with a fixable Live/Dead dye (Thermo Fisher Scientific) before incubation with saturating concentrations of surface mAbs diluted in 50% Brilliant stain buffer (BD Biosciences, San Jose, CA, USA) and 50% 1x PBS for 30 min at 4°C. Cells were fixed and permeabilized with Cytofix/Cytoperm (BD Biosciences) according to the manufacturer’s instructions. Intracellular staining was then performed by incubation of mAbs diluted in 0.1% saponin (Sigma-Aldrich) for a further 30 min at 4°C. All samples were acquired on a BD LSR Fortessa flow cytometer and analyzed using FlowJo v10 (BD Biosciences). A list of mAbs used for flow cytometry is provided in **Table S4**.

***Statistical analysis***

Categorical variables are expressed as n (%) and quantitative variables as median and interquartile range (IQR). A Chi Square test was used to analyze relationships between categorical variables. For quantitative variables, paired comparisons were performed using a Wilcoxon Signed-Rank test and unpaired comparisons using a Mann-Whitney test, Welch’s t-test or Kruskall-Wallis test, when appropriate as indicated in the figure legends. For correlative analysis between two quantitative variables a Spearman’s Rank correlation coefficients test was used. The Kaplan-Meyer analysis was used to define the probability of functional cure, defined as HBsAg loss and the differences between groups. Statistical analyses were performed using either SPSS version 20.0 (IBM, Chicago, IL, USA), GraphPad Prism version 8.1 (GraphPad Software, Inc., CA, USA) or R version 3.4 (R foundation for statistical computing, Vienna, Austria).

Table S1**. Individual patient characteristics at baseline, 12 weeks, 1 year and 2 years after NA interruption (n=27 patients).**

| ID | Gender  /age | Outcome |  | Baseline^1^ |  |  |  | Week 12 |  |  | 1 Year | | | | |  | 2 Years | | | | ALT peak |
| --- | --- | --- | --- | --- | --- | --- | --- | --- | --- | --- | --- | --- | --- | --- | --- | --- | --- | --- | --- | --- | --- |
|  |  |  | **qHBsAg** | **HBcrAg** | **HBV-RNA** | **qHBsAg** | **HBcrAg** | **HBV-RNA** | **ALT** | **HBV-DNA** | **qHBsAg** | **HBcrAg** | **HBV-RNA** | **ALT** | **HBV-DNA** | **qHBsAg** | **HBcrAg** | **HBV-RNA** | **ALT** | **HBV-DNA** |  |
| 6 | M/60 | HBsAg loss | <0.13 | 0 | 0 | 0 | 0 | 120 | 20 | 208 | 0 | 0 | 0 | 20 | 0 | 0 | 0 | 0 | 16 | 0 | 25 |
| 8 | M/65 | HBsAg loss | 74 | 0 | 0 | 4 | 0 | 0 | 26 | 10 | 0 | 0 | 0 | 16 | 0 | 0 | 0 | 0 | 13 | 0 | 72 |
| 9 | M/77 | HBsAg loss | <0.13 | 0 | 0 | 0 | 0 | 0 | 16 | 10 | 0 | 0 | 0 | 17 | 0 | 0 | 0 | 0 | 17 | 0 | 17 |
| 11 | M/57 | HBsAg loss | 567 | 2.8 | 0 | 316 | 0 | 0 | 20 | 16 | 31 | 0 | 0 | 18 | 10 | 0 | 0 | 0 | 20 | 0 | 30 |
| 15 | M/60 | HBsAg loss | 329 | 0 | 0 | 345 | 4.5 | 6359 | 754 | 191000 | 0 | 0 | 0 | 16 | 0 | 0 | 0 | 0 | 29 | 0 | 754 |
| 18 | M/32 | HBsAg loss | 52 | 5.1 | 61 | 1561 | 6.9 | 4185 | 750 | 116000 | 0 | 4.10 | 0 | 19 | 15 | 0 | 4.20 | 0 | 28 | 0 | 750 |
| 19 | F/59 | HBsAg loss | 1755 | 0 | 0 | 32 | 0 | 0 | 32 | 0 | 0 | 0 | 0 | 14 | 0 | 0 | 0 | 0 | 11 | 0 | 535 |
| 26 | M/60 | HBsAg loss | 66 | 0 | 0 | 40 | 0 | 1840 | 69 | 2360 | 1 | 0 | 0 | 21 | 61 | 0 | 0 | 0 | 21 | 0 | 69 |
| 1 | M/57 | Viral control | 6069 | 3.1 | 58 | 7290 | 2.8 | 78 | 44 | 982 | 5062 | 3.20 | 760 | 30 | 22900 | 4824 | n.a. | 0 | 30 | 26800 | 132 |
| 3 | M/43 | Viral control | 1260 | 3.0 | 0 | 112 | 3.2 | 0 | 32 | 10 | n.a. | 0 | 0 | 37 | 10 | 6 | 0 | 0 | 33 | 10 | 98 |
| 4 | M/56 | Viral control | 598 | 0 | 0 | 405 | 0 | 0 | 49 | 88 | 300 | 0 | 102 | 26 | 450 | 372 | 0 | 526 | 30 | 567 | 419 |
| 5 | M/41 | Viral control | 638 | 3.0 | 0 | 373 | 3.1 | 0 | 37 | 174 | 298 | 0 | 0 | 26 | 85 | 86 | 0 | 0 | 25 | 105 | 241 |
| 14 | M/38 | Viral control | 1569 | 0 | 0 | 2018 | 0 | 0 | 49 | 421 | 1909 | 0 | 0 | 33 | 4820 | 1668 | 0 | 0 | 35 | 7000 | 94 |
| 16 | M/43 | Viral control | 4145 | 2.8 | 96 | 2799 | 0 | 0 | 53 | 353 | 2883 | 0 | 0 | 32 | 3700 | 2446 | 0 | 898 | 30 | 6680 | 141 |
| 17 | F/47 | Viral control | 3495 | 3.1 | 97 | 4774 | 3.0 | 127 | 14 | 226 | 3478 | 3.30 | 5236 | 31 | 29800 | 780 | 2.90 | 1319 | 15 | 404 | 57 |
| 20 | M/55 | Viral control | 23466 | 0 | 0 | 20771 | 0 | 0 | 22 | 1950 | 10519 | 3.50 | 105 | 21 | 5770 | 8727 | 3.10 | 108 | 20 | 17900 | 71 |
| 21 | F/51 | Viral control | 2365 | 3.1 | 136 | 3138 | 0 | 85 | 39 | 1410 | 1689 | 3.40 | 4421 | 33 | 22000 | 1715 | n.a. | 3450 | 34 | 11900 | 85 |
| 22 | M/48 | Viral control | 3031 | 3.3 | 818 | 3217 | 2.8 | 508 | 90 | 5670 | 1862 | 0 | 1910 | 36 | 12400 | 1817 | 0 | 158 | 60 | 9500 | 80 |
| 23 | M/51 | Viral control | 1675 | 0 | 889 | 1802 | 3.1 | 2402 | 23 | 6580 | 375 | 0 | 0 | 25 | 1240 | 115 | 0 | 0 | 21 | 93 | 169 |
| 24 | M/73 | Viral control | 583 | 0 | 0 | 631 | 0 | 0 | 26 | 14 | 450 | 0 | 0 | 25 | 52 | 153 | 0 | 0 | 23 | 450 | 50 |
| 25 | F/65 | Viral control | 1240 | 2.9 | 101 | 1137 | 3.1 | 0 | 12 | 10 | 743 | 3.0 | 718 | 16 | 11000 | 859 | n.a. | n.a. | 17 | 5830 | 54 |
| 27 | F/54 | Viral control | 5012 | 0 | 0 | 4166 | 0 | 0 | 16 | 31 | 3478 | 0 | 0 | 31 | 29800 | 3722 | 0 | 0 | 36 | 177 | 56 |
| 2 | M/68 | NA reintrod ^2^ | 2530 | 3.0 | 1002 | 30212 | 6.7 | -- | -- | -- | -- | -- | -- | -- | -- | -- | -- |  | -- | -- | 519 |
| 7 | F/62 | NA reintrod ^2^ | 3786 | 3.4 | 842 | 4114 | 3.6 | 2192 | 16 | 136 | 7843 | 6.20 | 1314548 | 92 | 4200000 | -- | -- |  | -- | -- | 139 |
| 10 | M/44 | NA reintrod ^2^ | 2122 | 2.8 | 364 | 2306 | 3.6 | 5482 | 27 | 47500 | -- | -- | -- | -- | -- | -- | -- |  | -- | -- | 159 |
| 12 | M/46 | NA reintrod ^2^ | 1310 | 0 | 0 | 248 | 4.4 | 21184 | 518 | 904000 | -- | -- | -- | -- | -- | -- | -- |  | -- | -- | 518 |
| 13 | M/44 | NA reintrod ^2^ | 556 | 0 | 0 | -- | -- | -- | -- | -- | -- | -- | -- | -- | -- | -- | -- |  | -- | -- | 340 |

ID, patient identification; M, male, F, female; qHBsAg, quantitative HBsAg (IU/ml); HBcrAg (log U/ml); HBV-RNA, serum HBV-RNA (copies/ml); ALT, alanine aminotransferase (IU/L); HBV-DNA (IU/ml); n.a., not available.

^1^ Before NA discontinuation (baseline), all patients had undetectable HBV-DNA levels and normal ALT values. ALT peak shows maximum ALT value while off-therapy.

^2^ In patients with treatment reintroduction (NA reintrod), only data regarding qHBsAg, HBV-DNA and ALT levels off-therapy are shown; (--) indicating NA reintroduction. All patients with NA reintroduction had undetectable HBV-DNA and normal ALT (< ULN) at the end of follow-up.

Table S2**. Correlations between baseline serum and liver virological markers.**

|  | iHBV-DNA | cccDNA | iHBV-RNA^#^ | iHBV-RNA/cccDNA^#^ |
| --- | --- | --- | --- | --- |
| qHBsAg | rho= 0.65  p= 0.0003 | rho= 0.33  p= 0.09 | rho= 0.48  p= 0.04 | rho= 0.45  p= 0.06 |
| serum HBV-RNA | rho= 0.50  p= 0.008 | rho= 0.22  p= 0.3 | rho= 0.56  p= 0.01 | rho= 0.56  p= 0.01 |
| HBcrAg | rho= 0.36  p= 0.06 | rho= 0.18  p= 0.4 | rho= 0.34  p= 0.2 | rho= 0.24  p= 0.3 |

rho, Spearman’s correlation coeficient; p, p-value; qHBsAg, quantitative HBsAg; iHBV-DNA, intrahepatic HBV-DNA; cccDNA, covalently closed circular DNA; iHBV-RNA, intrahepatic HBV-RNA

^#^ Data available for 18 of 27 patients.

Table S3**. Primers and probes for cccDNA and 3.5 kb HBV-RNA detection.^1^**

| Target | Primer and probe sequence (5’-3’) | | Nucleotide position^#^ |
| --- | --- | --- | --- |
| cccDNA | Forward | CCGTGTGCACTTCGCTTCA | 1577–1595 |
|  | Reverse | GCACAGCTTGGAGGCTTGA | 1884–1866 |
|  | Probe | CATGGAGACCACCGTGAACGCCC | 1609–1631 |
| HBV-RNA | Forward | GGTCCCCTAGAAGAAGAACTCCCT | 2363–2386 |
|  | Reverse | CATTGAGATTCCCGAGATTGAGAT | 2450–2427 |
|  | Probe | TCTCAATCGCCGCGTCGCAGA | 2404–2424 |

^#^ Reference to NCBI Reference Sequence: NC_003977.2.

Table S4**. Monoclonal antibody details.**

| Antigen | Fluorochrome | Manufacturer | Clone | Catalog No. |
| --- | --- | --- | --- | --- |
| *Phenotype* |  |  |  |  |
| CD3 | BUV805 | BD | UCHT1 | 565515 |
| CD4 | V500 | BD | RPA-T4 | 560768 |
| CD8 | Alexa Fluor 700 | ThermoFisher | OKT8 | 56-0086-42 |
| CD19 | FITC | Biolegend | SJ25C1 | 363008 |
| CD56 | BV605 | Biolegend | HCD56 | 318334 |
| Live/Dead | APC-Cy7 | ThermoFisher |  | L10119 |
| *Function* |  |  |  |  |
| CD107a | Pacific Blue | Biolegend | H4A3 | 328624 |
| IFNɣ | PerCP-Cy5.5 | Biolegend | 4S.B3 | 502525 |
| TNFα | PE-Dazzle | Biolegend | MAb11 | 502946 |

Fig. S1**. HBsAg kinetics prior to NA discontinuation.** HBsAg levels in available stored serum samples, obtained up to 24 months before stopping NA therapy. Patients that achieved functional cure after NA discontinuation are shown in blue.

Fig. S2**. Occurrence of HBsAg loss after NA discontinuation.** Kaplan-Meier curves for HBsAg loss after NA interruption according to HBsAg levels and the presence or absence of serum HBV-RNA at baseline: HBsAg ≤1000 IU/ml and undetectable HBV-RNA *vs.* HBsAg >1000 IU/ml or detectable HBV-RNA.


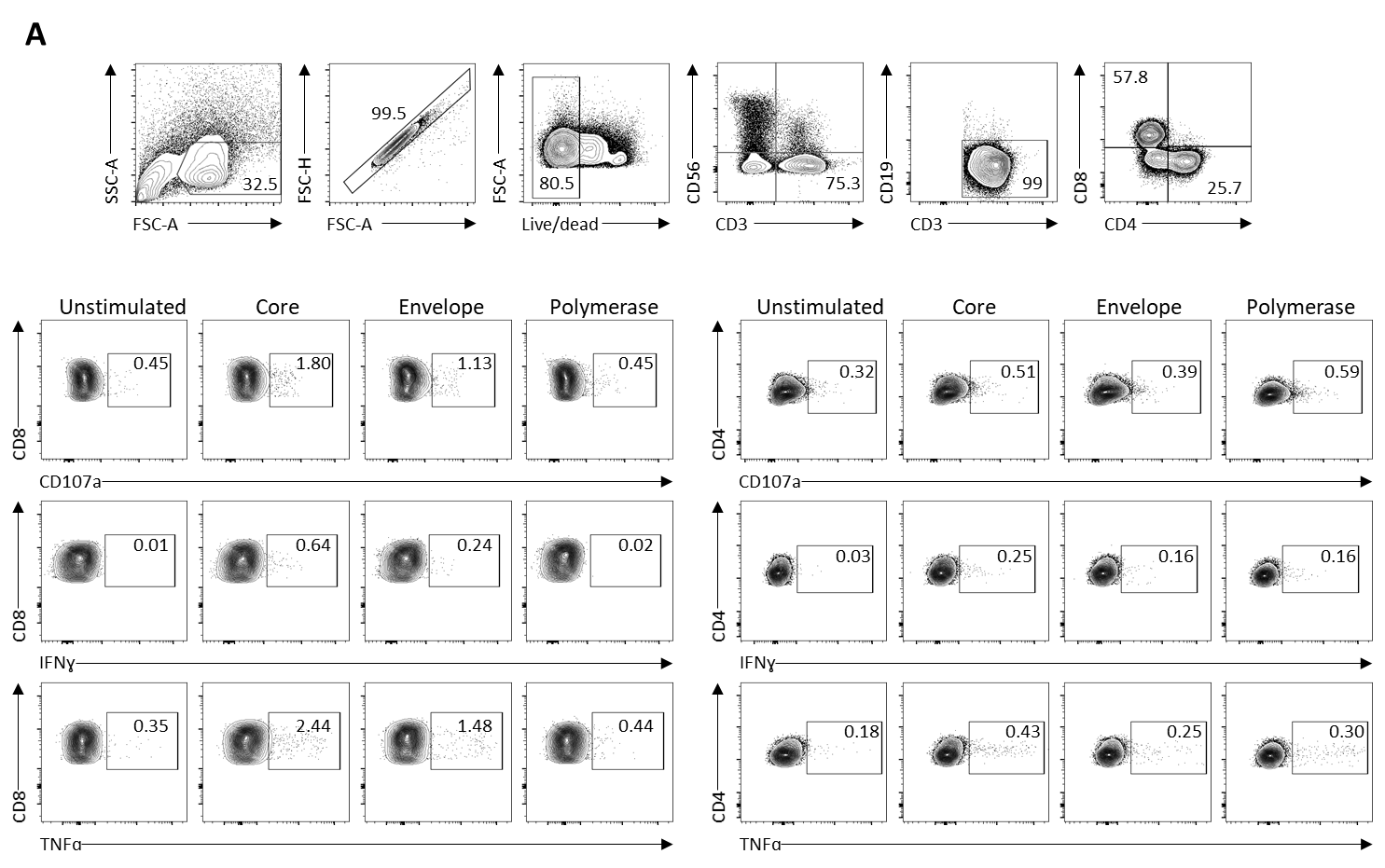


Fig. S3. **Flow cytometry gating strategy.** Representative plots identifying peripheral CD8+ and CD4+ T cells and HBV-specific T cell responses after stimulation with HBV OLP encompassing core, envelope and polymerase proteins.

1. **CD8+ T cells** Off-therapy patients Re-treated patients

2. **CD4+ T cells** Off-therapy patients Re-treated patients


Fig. S4**. HBV-specific T cell responses at baseline and during follow-up.** The percentage of HBV-specific CD8+ (A) and CD4+ T (B) cells expressing CD107a, IFNγ or TNFα was assessed after *in vitro* stimulation with HBV OLP spanning the core, envelope (Env), or polymerase (Pol) proteins at baseline (BL) and at week 12 (W12) and 48 (W48) after treatment discontinuation.

Fig. S5**. Degranulating CD8+ T cells at baseline and during follow-up.** Percentage of CD8+ T cells expressing CD107a after *in vitro* expansion in the presence of HBV OLP encompassing the envelope (Env) and polymerase (Pol) proteins at baseline and 48 weeks after stopping treatment. Bars represent the median and dotted lines the 0.1% cut-off for positive T cell responses minus the unstimulated control. Welch’s t-test was used to compare groups. No differences were observed between patients that remained off-therapy and those patients needing treatment reintroduction.

Fig. S6**. Polyfunctional HBV-specific T cells at baseline and during follow-up.** Percentage of CD8+ and CD4+ T cells co-expressing IFNɣ and TNFɑ after *in vitro* expansion in the presence of HBV OLP encompassing the envelope (Env) and polymerase (Pol) proteins at baseline and 48 weeks after treatment withdrawal. Bars represent the median. Welch’s t-test was used to compare groups. No differences were observed between patients that remained off-therapy and those patients needing treatment reintroduction.

Fig. S7**. Baseline HBV-specific T cell responses according to ALT flares during follow-up.** Upon stopping NA therapy, patients were classified according to the presence or absence of clinically relevant ALT flares during follow-up. The percentage of baseline HBV-specific CD8+ or CD4+ T cells expressing CD107a, IFNγ or TNFα was assessed after *in vitro* stimulation with HBV OLP spanning the core, envelope, or polymerase proteins.

**A**

**B**

Fig. S8**. HBV-specific T cell responses according to HBsAg levels at baseline.** (A) Percentage of patients with baseline HBV-specific CD8+ or CD4+ T cell responses after *in vitro* HBV OLP stimulation. Patients were classified according to HBsAg levels ≤1000 IU/ml or >1000 IU/ml. The number of patients is shown within the bars. Pie charts below the bars represent the fraction of patients with T cell responses against the different HBV OLP pools: Core (dark blue), Env (mid blue) and Pol (light blue). (B) Polyfunctional HBV-specific CD8+ and CD4+ T cells co-expressing IFNγ or TNFα after core OLP stimulation at baseline and 48 weeks after NA treatment discontinuation. Bars represent the median.

References

1. Malmström, S., Larsson, S. B., Hannoun, C., Lindh, M. Hepatitis B viral DNA decline at loss of HBeAg is mainly explained by reduced cccdna load - down-regulated transcription of PgRNA has limited impact. PLoS One. 2012;7(7):e36349.
2. Tong, Y., Liu B, Liu H, Zheng H, Gu J, Liu H, et al. New universal primers for genotyping and resistance detection of low HBV DNA levels. Medicine (Baltimore). 2016;95(33):e4618.
3. Larkin MA, Blackshields G, Brown NP, Chenna R, McGettigan PA, McWilliam H,. et al. Clustal W and Clustal X version 2.0. Bioinformatics. 2007;23(21):2947-8.
4. Kumar, S., Stecher, G., Li, M., Knyaz, C., Tamura, K. MEGA X: Molecular Evolutionary Genetics Analysis across Computing Platforms. Mol Biol Evol. 2018;35(6):1547-1549.
5. Allweiss L, Volz T, Giersch K, Kah J, Raffa G, Petersen J, et al. Proliferation of primary human hepatocytes and prevention of hepatitis B virus reinfection efficiently deplete nuclear cccDNA in vivo. Gut. 2018;67(3):542-552.)
