# Supplementary material for: Viral and immune factors associated with successful treatment withdrawal in HBeAg-negative chronic hepatitis B patients
Source: J Hepatol. 2021 May;74(5):1064–74. doi: 10.1016/j.jhep.2020.11.043 (PMC8062913; doi:10.1016/j.jhep.2020.11.043)
Supplement: Multimedia component 2 [file mmc2.pdf]

JOURNAL
OF HEPATOLOGY

Journal of Hepatology

CTAT methods

Tables for a "Complete, Transparent, Accurate and Timely account" (CTAT) are now mandatory for all revised submissions. The aim is to enhance the reproducibility of methods.

- Only include the parts relevant to your study
- Refer to the CTAT in the main text as 'Supplementary CTAT Table'
- Do not add subheadings
- Add as many rows as needed to include all information
- Only include one item per row

If the CTAT form is not relevant to your study, please outline the reasons why:

All this information is included in the manuscript as supplementary data.

1.1 Antibodies

Name	Citation	Supplier	Cat no.	Clone no.
CD3		BD	565515	UCHT1
CD4		BD	560768	RPA-T4
CD8		ThermoFisher	56-0086-42	OKT8
CD19		Biolegend	363008	SJ25C1
CD56		Biolegend	318334	HCD56
Live/Dead		ThermoFisher	L10119	
CD107a		Biolegend	328624	H4A3
IFNɣ		Biolegend	502525	4S.B3
TNFα		Biolegend	502946	MAb11

1.2 Sequence based reagents

Name	Sequence	Supplier
HBV overlapping peptides (pangenotypic or genotype D)	On request	Gilead & Peptide Synthesis Core Facility, UPF
ProMix™ CEF Peptide Pool	PX-CEF	Proimmune
Human b-globin assay	Hs00758889_s1	Thermo Fisher
Total HBV DNA assay	Pa03453406_s1	Thermo Fisher
iHBV-RNA forward	GGTCCCCTAGAAGAAGAACTCCCT	Biomers
iHBV-RNA reverse	CATTGAGATTCCCGAGATTGAGAT	Biomers
iHBV-RNA probe	TCTCAATCGCCGCGTCGCAGA	Biomers
cccDNA forward	CCGTGTGCACTTCGCTTCA	Biomers
cccDNA reverse	GCACAGCTTGGAGGCTTGA	Biomers
cccDNA probe	CATGGAGACCACCGTGAACGCCC	Biomers
HBV_RT_F	TTCTTAGCGTATTGGAGCTC<br>CTGCTGGTGGCTCCAGTT	Biomers
HBV_RT_R	AAGTATCAGTATTGCGTTGTT<br>GCTAGGAGTTCCGCAGTATGG	Biomers
HBV_RT_F2	TTCTTAGCGTATTGGAGCTC	Biomers
HBV_RT_R2	AAGTATCAGTATTGCGTTGTT	Biomers
Internal RNA control assay	Int-RNA-VIC	Primer Design

1.3 Biological samples

Description	Source	Identifier
Serum samples<br>Liver biopsies<br>Peripheral blood mononuclear cells	Liver Unit, Hospital Clínic Barcelona, Spain	

1.4 Software

Software name	Manufacturer	Version
FlowJo	BD Bioscience	10
R	The R Foundation for Statistical Computing,	3.4
MEGA X	MEGA X	10
GraphPad Prism	GraphPad Software, Inc	8.1
SPSS	IBM	20

1.5 Please provide the details of the corresponding methods author for the manuscript:

Sofía Pérez-del-Pulgar
Liver Unit, Hospital Clínic, IDIBAPS, Barcelona, Spain.
Tel.: +34 2275400, ext. 2093.
Fax: +34 933129405.
E-mail address: sofiapp@clinic.cat
